# Development of a Prognostic Nomogram in Epithelial Ovarian Cancer Based on KELIM: A Retrospective Study at TuDu Hospital, Vietnam

**DOI:** 10.3390/diagnostics16010151

**Published:** 2026-01-02

**Authors:** Hoang T. Pham, Tuan M. Vo, Le N. N. Phan, Hien T. Nguyen

**Affiliations:** 1 Medicine Faculty, University of Medicine and Pharmacology at Ho Chi Minh City, Ho Chi Minh City 7000, Vietnam; drthanhhoangpham@ump.edu.vn; 2 TuDu Hospital, Ho Chi Minh City 7000, Vietnam

**Keywords:** epithelial ovarian cancer, KELIM, nomogram, survival time

## Abstract

**Background/Objectives:** Epithelial ovarian cancer (EOC) constitutes the predominant form of ovarian malignancies. The primary goal of this study was to determine predictors of patient survival and construct a nomogram for survival prediction in individuals diagnosed with epithelial ovarian cancer. **Methods:** A retrospective cohort analysis was performed, including 418 patients who received treatment for epithelial ovarian cancer at Tu Du Hospital from January 2015 to December 2019. The median follow-up time was 77.1 months (range: 5.7–121.6 months). Survival analyses were conducted using the log-rank test and Cox proportional hazard regression analysis. A nomogram was developed, incorporating KELIM and other statistically significant variables. **Results:** The median follow-up time was 77.1 months. The observed cumulative mortality rates were 1.4% (95% confidence interval [CI]: 0.7–3.2), 10.4% (95% CI: 7.8–13.8), and 16.5% (95% CI: 13.2–20.6) at 1, 3, and 5 years, respectively. Factors demonstrating a significant correlation with survival included KELIM < 1 (HR = 1.78 [95% CI: 1.16–2.72]), pre-treatment CA-125 levels ≥ 35 U/mL (HR = 2.47 [95% CI: 1.10–5.55]), FIGO stages III-IV (HR = 2.40 [95% CI: 1.36–4.21]), and the presence of residual tumor tissue following surgical intervention (HR = 3.14 [95% CI: 1.75–5.65]). **Conclusions:** Prognosis is significantly influenced by KELIM, pre-treatment CA-125, tumor stage, and residual tumor post-surgery. The nomogram developed here offers a tool to assist in personalized prognostic assessments of Vietnamese EOC patients.

## 1. Introduction

Epithelial ovarian cancer (EOC) is the predominant form of ovarian malignancy, accounting for 82% to 91% of all diagnoses [1]. The disease frequently manifests with a lack of early symptoms, resulting in approximately 70% of patients being diagnosed at an advanced stage (FIGO stage III-IV) [1], subsequently leading to a poor prognosis for survival. Global statistics from GLOBOCAN for 2022 documented 324,603 new occurrences and 206,956 fatalities stemming from ovarian cancer [2]. In Vietnam, in the same year, 1404 new diagnoses and 923 deaths were attributed to this disease [2].

Although therapeutic interventions have improved, the 5-year survival rate for EOC remains limited (35–57%), and the recurrence rate is elevated (>95%) [1,3]. Consequently, the identification of early and accurate prognostic indicators is critical for tailoring treatment strategies, thus enhancing both patient survival and overall quality of life.

Numerous established prognostic factors impact survival, including cancer stage, the amount of residual tumor following debulking surgery, and initial levels of cancer antigen (CA-125) [4,5]. More recently, the CA-125 Elimination rate constant K (KELIM), introduced by You et al. in 2013, has emerged as a dynamic marker that reflects the decrease in CA-125 levels during chemotherapy [6]. KELIM is a model-based kinetic parameter derived from the early longitudinal decline of serum CA-125 during first-line platinum-based chemotherapy and is considered an early surrogate of intrinsic tumor chemosensitivity. Practically, KELIM is estimated using at least three CA-125 measurements obtained during the first three chemotherapy cycles (within approximately the first 100 days after chemotherapy initiation), applying a validated population kinetic model implemented in an online calculator (Biomarker Kinetics™, was developed by a research team from Lyon University (Université Claude Bernard Lyon 1, France) and Lyon University Hospital (Hospices Civils de Lyon, France)). Higher KELIM values indicate faster CA-125 elimination and have been consistently associated with improved outcomes in epithelial ovarian cancer KELIM indicates an early response to chemotherapy and demonstrates a significant association with the survival duration of patients with EOC [6,7].

Tu Du Hospital, the foremost obstetrics and gynecology referral center in Southern Vietnam, manages approximately 250 ovarian cancer cases annually. Notably, the institution operates under a distinct referral framework, wherein patients initially diagnosed with advanced-stage epithelial ovarian cancer are routinely directed to tertiary oncology centers for comprehensive multimodal management. Consequently, the majority of ovarian cancer patients treated at Tu Du Hospital are diagnosed at an early stage and are typically suitable candidates for primary radical cytoreductive surgery. This institutional treatment model accounts for the predominance of early-stage disease in our study cohort. Despite the center’s high clinical volume, KELIM has not seen widespread application in early survival predictions at this facility. This research initiative aimed to assess the utility of KELIM and its associated elements in forecasting the survival outcomes of patients with EOC.

## 2. Materials and Methods

A retrospective cohort analysis was performed between June 2024 and March 2025, focusing on individuals diagnosed with epithelial ovarian cancer (EOC) treated at Tu Du Hospital, with initial surgical interventions occurring between January 2015 and December 2019. This study encompassed the entire available population within a specified timeframe. All cases confirmed with a histopathological diagnosis of EOC and subjected to a minimum of three cycles of carboplatin-paclitaxel chemotherapy constituted the inclusion criteria. Patients with concurrent malignancies or incomplete medical records were excluded from the study. The research protocol was approved by the Institutional Review Board of Tu Du Hospital (Approval Number: 610/QD-BVTD, 9 April 2024). Patient consent was waived due to the retrospective nature of the study.

The data collected included basic demographic information, pretreatment CA-125, cancer stage (FIGO 2021) [8], number of chemotherapy cycles, and surgical and pathological characteristics. KELIM was calculated using the website “Circulating tumor biomarker—Biomarker Kinetics™” (accessed on 4 December 2025) using CA-125 levels in the first three chemotherapy cycles. The KELIM was divided into two groups: favorable (KELIM ≥ 1) and unfavorable (KELIM < 1) [9,10]. Initial treatment response was defined according to the RECIST 1.1 criteria [11]. Three weeks after the end of the last chemotherapy cycle, patients were scheduled for follow-up visits to assess residual tumor tissue using imaging and CA-125 test results. Patients were defined as having responded to treatment when there was no residual tumor tissue after treatment and CA-125 was <35 U/mL; Patients did not respond to treatment when tumor tissue remained after treatment or CA-125 was ≥35 U/mL. Follow-up imaging was performed according to the treating physician’s clinical indication. In our institution, post-treatment assessment of residual disease was primarily conducted using pelvic/abdominal ultrasound, with pelvic MRI utilized when additional anatomic characterization was required or when ultrasound findings were equivocal. Imaging results were interpreted in conjunction with clinical evaluation and serum CA-125 levels to determine treatment response and the presence of residual tumor.

Time from surgery to start of chemotherapy (TTC) was defined as the interval (in days) from the date of surgery to the date of initiation of first adjuvant chemotherapy. The neutrophil-to-lymphocyte ratio (NLR) and platelet-to-lymphocyte ratio (PLR) were categorized using optimal thresholds identified via receiver operating characteristic (ROC) curve analysis, achieving 90% sensitivity in predicting 5-year mortality (NLR: 1.61; PLR: 114.43). Survival duration, measured in months, was calculated from the date of diagnosis to either all-cause mortality [12] the most recent documented survival date, or the study’s conclusion in March 2025.

Calculating sample size with survival research formula:

$$n\;=\;{2(Z_{1-\frac{\alpha }{2}}+Z_{1-\beta })}^{2}/{\left(lnHR\right)}^{2}$$

α = 0.05; β = 0.1. Pauline Corbaux’s 2023 systematic review study [7] found that the group with favorable KELIM had a longer progression-free survival than the unfavorable KELIM group with HR = 0.49. Substituting this into the formula, n = 98 was obtained as the minimum sample size to ensure sample power for the main objective.

**Statistical Analysis:** Data analysis was performed utilizing the Stata 17.0 software (Stata Corp LLC, Lakeway Drive College Station, College Station, TX, USA, April 2021). Descriptive statistics for the study cohort are presented using proportions or medians. The cumulative survival rate was calculated using the life table method. Between-group survival differences were assessed using the log-rank test. Univariate Cox regression models were implemented, followed by multivariate Cox regression models to identify factors correlated with mortality. Variables exhibiting a *p*-value less than 0.2 in the univariate analysis were incorporated into the multivariate Cox regression model. Statistical significance was set at a *p*-value of 0.05.

A nomogram model was constructed based on the statistically significant variables identified in the multivariate Cox regression. Pearson’s correlation coefficient was used to exclude highly correlated variables. A stepwise forward selection method was used, with a significance threshold of *p* < 0.05. Following the standardization of the regression coefficients, each patient’s total score was utilized to estimate the survival probability at 1, 3, and 5 years. The predictive performance of the nomogram was primarily assessed in terms of discrimination. We reported Harrell’s concordance index (C-index) and the corresponding Somers’ D statistic. In addition, time-specific discrimination was evaluated using receiver operating characteristic (ROC) analysis, and the areas under the curve (AUCs) were calculated at 1, 3, and 5 years.

## 3. Results

In total, 418 eligible patients were recruited. At the end of the study, 89 patients died, 215 were alive, and 114 were censored. The clinical characteristics of the patients are summarized in Table 1. The median age of the patients was 49 years (range: 16–74). More than 75% of the patients had CA-125 level of ≥35 U/mL. Nearly two-thirds of the patients had favorable KELIM results. More than three-quarters of the patients were diagnosed at an early stage (FIGO stage I-II). Most patients (90%) underwent primary debulking surgery (PDS) and radical surgery. The response rate to the primary treatment was very high (93.8%). The median time from surgery to initiation of adjuvant chemotherapy (TTC) was 20 days (IQR 7; range 5–229), consistent with our institutional 3-week scheduling protocol.

The median follow-up time was 77.1 months (range: 5.7–121.6 months). The cumulative mortality rates at 1, 3, and 5 years were 1.4% [95% CI: 0.7–3.2], 10.4% [95% CI: 7.8–13.8], and 16.5% [95% CI: 13.2–20.6] respectively (Table 2).

Ten covariates that demonstrated a significant association with survival were identified using the univariate Cox regression analysis. To maintain model stability and prevent overfitting, the subsequent multivariate model’s variable selection adhered to the guidelines of a minimum of ten events per predictor variable. Given the observed 89 instances of mortality, a set of eight variables was incorporated into the multivariate model. To preserve the model’s ability to offer early prognostic information, variables pertaining to treatment response and total chemotherapy cycles were excluded, as the determination of these factors necessitates completion of the primary treatment regimen.

Multivariate Cox model results showed that factors increasing the risk of death included unfavorable KELIM, HR = 1.78 [95% CI: 1.16–2.72]; pre-treatment CA-125 ≥ 35 U/mL, HR = 2.47 [95% CI: 1.10–5.55]; advanced-stage cancer (FIGO III-IV), HR = 2.40 [95% CI: 1.36–4.21]; and residual tumor tissue after debulking surgery, HR = 3.14 [95% CI: 1.75–5.65] (Table 3).

**Figure 1:**
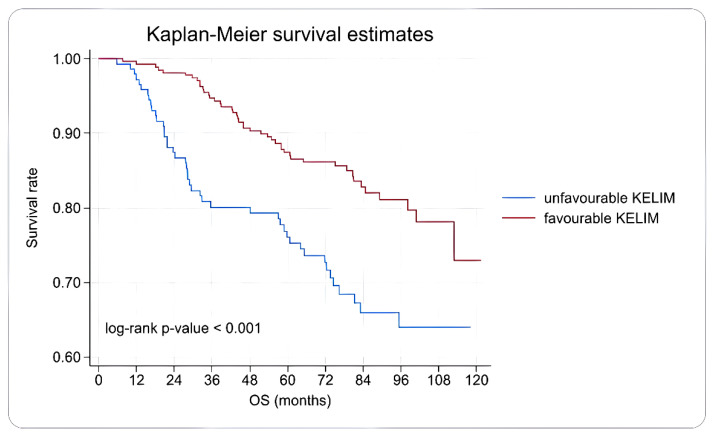
Kaplan–Meier overall survival curve according to KELIM score.

Overall survival in the favorable KELIM group was better than that in the unfavorable KELIM group. This difference was statistically significant, with *p* < 0.001 (log-rank test). At 1 year, the survival rates in the favorable KELIM group and the unfavorable KELIM group were 99.3% and 97.2%, respectively. At 3 years, the survival rates in the favorable KELIM group and the unfavorable KELIM group were 94.7% and 80.1%, respectively. At 5 years, the survival rates in the favorable KELIM group and the unfavorable KELIM group were 87.4% and 76.1%, respectively (Figure 1).

**Figure 2:**
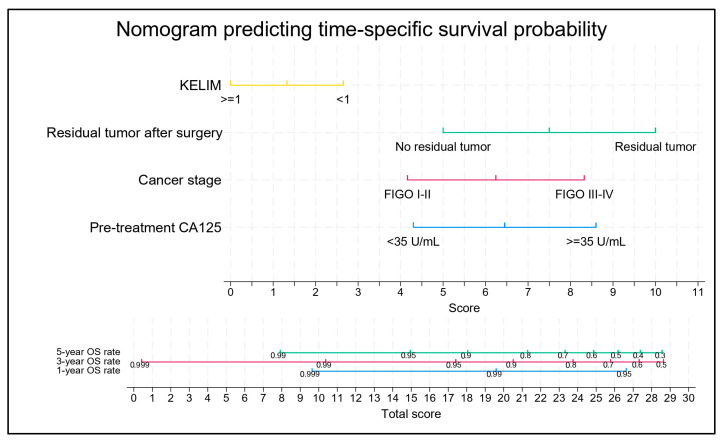
Nomogram predicting time-specific survival probability.

Pearson’s correlation coefficients were computed for factors exhibiting significant associations in the multivariate Cox regression model. These analyses revealed negligible collinearity among the identified predictors. Employing a stepwise forward selection strategy, all variables demonstrated statistical significance, with *p*-values below 0.05. Consequently, these four contributing factors were incorporated into the multivariate Cox regression model, which was subsequently utilized in the nomogram (Figure 2). The predictive performance of the model was evaluated using Harrell’s C-index and Somers’ D statistic. The C-index was 0.7628 and the associated Somers’ D value was 0.5256. These findings suggest a noteworthy ability to differentiate patients based on their survival prospects.

## 4. Discussion

The modeled KELIM has been established as a dynamic marker of intrinsic chemosensitivity derived from early CA-125 kinetics during platinum-based chemotherapy. Across multiple datasets, KELIM has shown consistent prognostic value for progression-free survival and overall survival, supporting its role as an early, treatment-informed biomarker beyond static baseline variables [6,7,10,13]. In addition to survival prognostication, KELIM has been investigated for clinically actionable applications in treatment planning. Several studies in advanced-stage disease treated with neoadjuvant chemotherapy have reported that favorable KELIM is associated with a higher probability of achieving complete interval debulking surgery [14], suggesting potential value as a triage tool to anticipate surgical outcomes. Moreover, exploratory trial-based analyses have evaluated KELIM as a complementary biomarker for treatment selection, including identifying subgroups more likely to benefit from PARP inhibitor strategies [15], highlighting its potential role in contemporary maintenance decision frameworks.

The median KELIM value observed in this investigation was 1.1, with a range spanning from 0.3 to 2.3. The incidence of unfavorable KELIM results was 34.5%, which was demonstrably lower than that reported in previous research, which varied between 47.2% and 67.4% [3,7,16]. Among advanced cancer in this study, the unfavorable KELIM rate was 41.8%. This figure aligns with the range of 19.0–58.1% found in alternative publications [17,18]. A lack of uniformity characterized the distribution of favorable and unfavorable KELIM rates across the reviewed literature. Nevertheless, a shared observation across several investigations, including this one, was the predominance of favorable KELIM outcomes. This indicates that the majority of the patients exhibited a positive response to chemotherapy. This finding corroborates with our study’s robust treatment response rate of 93.8%.

The high proportion of early-stage disease and optimal cytoreduction observed in our study reflects institutional referral patterns. At Tu Du Hospital, advanced-stage patients with high tumor burden or anticipated surgical complexity are commonly referred to tertiary oncology centers for specialized treatment. This referral model inherently selects for patients suitable for primary surgery, contributing to favorable baseline characteristics. Our findings are consistent with those reported by Nguyen et al. (2022) [19], who also documented a predominance of early-stage EOC in a cohort treated at the same institution. This alignment reinforces the internal validity of our stage distribution and highlights the systematic influence of our center’s treatment policy. Nevertheless, this selection bias may amplify the apparent prognostic strength of tumor biology markers such as KELIM. Therefore, caution should be exercised when extrapolating our results to broader or more advanced-stage EOC populations, where survival is more critically influenced by surgical feasibility and perioperative factors.

The relatively long median follow-up (77.1 months) strengthens the reliability of the reported 5-year overall survival estimates. The 1-year, 3-year, and 5-year survival rates in our study were 98.6, 89.6, and 83.5%, respectively. The survival rates in our study were higher than those reported in other countries. The US ovarian cancer statistics (2018) showed that the 5-year survival rate of EOC in Asian/Pacific Islanders is 57% [1]. A UK study (2022) showed that the 1-year and 5-year survival rates were 76% and 38%, respectively [20]. A Chinese study by Bai et al. (2023) showed that the 1-year, 3-year, and 5-year survival rates of 76%, 59%, and 45%, respectively [21]. In the early-stage cancer group, the 5-year survival rate was 90.4%. This rate is comparable to that of some studies, with 5-year survival rates ranging from 89.8% to 94.5% [22,23]. The survival rates in our study were generally higher than those reported in previous studies. This difference may be because our study included a large number of patients diagnosed at an early stage (75.4%) who achieved optimal debulking surgery (91.9%).

Multivariate regression analysis identified four independent predictors of survival duration: KELIM level, pretreatment CA-125 concentration, cancer stage, and the presence of residual tumor tissue following surgical intervention. These prognostic indicators are consistent with the findings of extensive large-scale and multicenter investigations. [7,16,24,25] Consequently, the results of the present investigation align with established trends concerning factors associated with survival outcomes of patients with epithelial ovarian cancer (EOC).

Pretreatment CA-125 is an easily accessible biomarker that supports risk assessment and prognosis at the time of diagnosis. In addition to our study, other studies have also noted the prognostic role of CA-125 in survival, such as the pooled analysis of Wang et al. (2022) that recorded HR = 1.62 [95% CI: 1.27–2.06] [25]. However, some studies have denied the prognostic role of pretreatment CA-125 levels [26,27].

The cancer stage and residual tumor tissue after debulking surgery have a strong impact on survival. Patients diagnosed with advanced-stage disease have a 1.78- to 10-fold increased risk of death compared to those diagnosed with early-stage disease [7,16,28]. The difference in the impact of cancer stage on survival between studies may be due to differences in study populations or the choice of covariates for the multivariate Cox models. Optimal debulking surgery is an important goal in EOC treatment. Residual tumor tissue after surgery is a strong prognostic factor for survival [16,17,21,24]. Achieving a tumor-free postoperative course reflects surgical skill. It is also related to the cancer stage, histology, and response to preoperative chemotherapy.

Although residual disease status was included in the multivariate model as a surrogate for surgical outcome, we acknowledge that it may not fully capture the nuances of surgical effort or intraoperative complexity. At our institution, surgical procedures are comprehensively documented through standardized operative protocols; however, validated surgical complexity scoring systems—such as the Surgical Complexity Score [29]—were not routinely adopted during the study period. Consequently, while data on the extent of resection were available, the lack of a quantitative surgical complexity index limited our ability to include this variable in prognostic modeling. Future studies should incorporate formal scoring systems to evaluate the prognostic interaction between surgical effort, tumor biology, and survival outcomes.

The generally short TTC and limited variability in our cohort may partly explain the lack of association between TTC and overall survival, in contrast to advanced-stage cohorts where greater surgical burden and postoperative morbidity more frequently lead to treatment delays [30].

Postoperative morbidity and 30-day perioperative mortality were reviewed in our cohort. Complications were rare (two intraoperative bladder injuries with good recovery), and no 30-day deaths due to surgical complications were observed. This likely reflects the predominance of early-stage, surgically favorable cases, in contrast to advanced-stage cohorts characterized by higher operative burden and greater complication variability [31]; therefore, postoperative morbidity was not included as a covariate due to insufficient event frequency.

KELIM is a dynamic marker with increasingly recognized prognostic value [7,16,17,24]. Patients with favorable KELIM have a 44% to 54% lower risk of death than those with unfavorable KELIM [7,16]. Our study found that the unfavorable KELIM group had a 1.78-fold increased risk of death, which is similar to the study by Lazar et al. [17]. The consistency of KELIM across studies confirms the value of KELIM in accurate risk stratification, supporting prognosis and patient follow-up strategies.

The results of the nomogram score analysis reflected the impact of the following factors on survival prognosis: unfavorable KELIM (2.7 points), favorable KELIM (0.0 points), pre-treatment CA-125 ≥ 35 U/mL (8.6 points), CA-125 < 35 U/mL (4.3 points), advanced-stage cancer (8.3 points), early-stage cancer (4.2 points), residual tumor tissue after surgery (10.0 points), and no residual tumor tissue (5.0 points). For example, if a patient is diagnosed with EOC with a pretreatment CA-125 level of 50 U/mL, FIGO stage II, KELIM score of 1.2, and no residual tumor tissue after debulking surgery. The patient’s total score is 8.7 + 4.2 + 0.0 + 5.0 = 17.9 points. Referring to the chart, the patient’s 1-year, 3-year, and 5-year survival rates are 98%, 94%, and 90%, respectively.

We used the scores calculated from the nomogram model to calculate the scores for all patients in the study. The total score had a median of 17.8 points (range: 13.5–29.6). The areas under the ROC curves at 1, 3, and 5 years were 0.8617, 0.810, and 0.8023, respectively, indicating that the nomogram model had good discrimination ability. This confirms the practical value of the model for personalized survival prediction.

In our cohort, KELIM remained independently associated with overall survival and added prognostic information when integrated with key clinicopathologic factors (pretreatment CA-125, FIGO stage, and residual tumor postsurgery). Accordingly, the developed nomogram enables individualized estimation of 1-, 3-, and 5-year OS, which may support patient counseling, risk stratification, and follow-up planning in settings where molecular profiling (e.g., BRCA/HRD) is not routinely available.

**Limitations of study:** Several constraints were present within the scope of this investigation. Initially, the retrospective nature of the study design introduced the potential for inaccuracies stemming from data recording. Furthermore, the analysis did not include a separate examination of the individual histological subtypes of epithelial ovarian cancer (EOC). Third, our cohort was enriched for early-stage, surgically favorable EOC due to our institutional referral model, which may limit generalizability to advanced-stage populations. External validation in tertiary cancer centers is warranted. Fourth, our analysis did not account for maintenance therapies. During the study period (2015–2019), PARP inhibitors were not yet approved for clinical use in Vietnam and were not available in routine practice at our institution. Although bevacizumab was permitted, retrospective data on its administration were not consistently documented. Consequently, the survival model presented in this study reflects outcomes in the pre-maintenance era and may not capture survival improvements attributable to contemporary maintenance strategies. Lastly, the dataset from Tu Du Hospital lacked information regarding biomarkers, including BRCA1/2 and Homologous Recombination Deficiency (HRD) status, owing to site-specific clinical practices and treatment protocols. These markers are becoming increasingly critical for personalized treatment strategies and prognostic assessments.

## 5. Conclusions

The present investigation documented one-year, three-year, and five-year survival rates for EOC patients in Southern Vietnam, yielding figures of 98.6%, 89.6%, and 83.5%, respectively. Determinants significantly associated with patient survival included the KELIM score, pretreatment CA-125 levels, cancer stage, and the presence of residual tumor following cytoreductive surgery. To broaden the scope of the analysis, future research concerning EOC patients in Southern Vietnam will incorporate assessments of BRCA1/2 status and homologous recombination deficiency (HRD).

## Figures and Tables

**Table 1:** Clinical characteristics of EOC patients.

Characteristics	Total n (%)	Survived (n = 329) n (%)	Deaths (n = 89) n (%)	*p* Value *
Age				0.764
≤50	229 (54.8)	182 (43.5)	47 (11.2)	
>50	189 (45.2)	147 (35.2)	42 (10.0)	
Pre-treatment CA-125				<0.001
<35 U/mL	102 (24.4)	95 (22.7)	7 (1.7)	
≥35 U/mL	316 (75.6)	234 (60.0)	82 (19.6)	
Timing of cytoreductive surgery				0.004
PDS	395 (94.5)	315 (75.4)	80 (19.1)	
IDS	23 (5.5)	14 (3.3)	9 (2.2)	
Type of cytoreductive surgery				0.414
Radical	392 (93.8)	307 (73.4)	85 (20.3)	
Conservative	26 (6.2)	22 (5.3)	4 (1.0)	
Residual tumor after surgery				<0.001
No residual tumor	384 (91.9)	318 (76.1)	66 (15.8)	
Residual tumor	34 (8.1)	11 (2.6)	23 (5.5)	
Cancer stage				<0.001
Early stage	315 (75.4)	273 (65.3)	42 (10.0)	
Advanced stage	103 (24.6)	56 (13.4)	47 (11.2)	
Total number of chemotherapy cycles				<0.001
≤6 cycles	253 (60.5)	216 (51.7)	37 (8.9)	
>6 cycles	165 (39.5)	113 (27.0)	52 (12.4)	
KELIM				<0.001
≥1	274 (65.5)	229 (54.7)	45 (10.8)	
<1	144 (34.5)	100 (23.9)	44 (10.5)	
Treatment response				<0.001
Responder	392 (93.8)	325 (77.8)	67 (16.0)	
Non-responder	26 (6.2)	4 (1.0)	22 (5.3)	
Histopathological type				0.014
HGSC	113 (27.0)	81 (19.4)	32 (7.7)	
Others	305 (73.0)	248 (59.3)	57 (13.6)	
NLR				0.007
≤1.61	87 (20.8)	78 (18.7)	9 (2.2)	
>1.61	331 (79.2)	251 (60.0)	80 (19.1)	
PLR				0.002
≤114.43	86 (20.6)	78 (18.7)	8 (1.9)	
>114.43	332 (79.4)	251 (60.0)	81 (19.4)	
Time from surgery to start of chemotherapy				0.540
≤21 days	253 (60.5)	197 (47.1)	56 (13.4)	
>21 days	165 (39.5)	132 (31.6)	33 (7.9)	

* *p*-value from Log-rank test results; PDS: Primary debulking surgery; IDS: Interval debulking surgery; HGSC: High-grade serous carcinoma; KELIM: CA-125 ELIMination rate constant K; NLR: Neutrophil-to-lymphocyte ratio; PLR: Platelet-to-lymphocyte ratio.

**Table 2:** Cumulative mortality rate of EOC.

Time (Months)	Number of Patients at Beginning	Number of Deaths	Cumulative Mortality Rate (%)	95% CI
0	418	0	-	-
12	412	6	1.4	0.7–3.2
24	383	17	5.6	3.7–8.2
36	350	19	10.4	7.8–13.8
48	329	10	13.0	10.0–16.7
60	300	13	16.5	13.2–20.6
72	247	7	18.5	15.0–22.8
84	153	11	23.0	18.9–27.9
96	96	3	24.9	20.4–30.1
108	44	2	27.2	21.7–32.4
120	2	1	30.0	22.6–39.0

**Table 3:** Factors related to survival.

Factors	Risk Time (Month)	Deaths (n = 89)		Cox Proportional Hazard Regression: HR (95% CI)	
		n/Total	Incidence Rate (per 1000 Person-Month)	Univariate Analyses	Multivariate Analyses
Age					
≤50	16,578	47/229	2.84	1	
>50	13,824	42/189	3.04	1.07 (0.70–1.62)	
*p*-value				0.764	
Pre-treatment CA-125 level					
<35 U/mL	8250	7/102	0.85	1	1
≥35 U/mL	22,152	82/316	3.70	4.41 (2.04–9.55)	2.47 (1.10–5.55)
*p*-value				<0.001	0.028
Timing of cytoreductive surgery					
PDS	29,106	80/395	2.75	1	1
IDS	1296	9/23	6.94	2.65 (1.33–5.30)	1.32 (0.62–2.78)
*p*-value				0.006	0.471
Type of cytoreductive surgery					
Radical	28,383	85/392	2.99	1	
Conservative	2019	4/26	1.98	0.66 (0.24–1.80)	
*p*-value				0.417	
Residual tumor after surgery					
No residual tumor	29,030	66/384	2.27	1	1
Residual tumor	1373	23/34	16.75	8.26 (5.09–13.40)	3.14 (1.75–5.65)
*p*-value				<0.001	<0.001
Cancer stage					
Early stage	24,495	42/315	1.71	1	1
Advanced stage	5908	47/103	7.96	4.88 (3.20–7.44)	2.40 (1.36–4.21)
*p*-value				<0.001	0.002
Total number of chemotherapy cycles					
≤6 cycles	18,878	37/253	1.96	1	
>6 cycles	11,525	52/165	4.51	2.32 (1.52–3.54)	
*p*-value				<0.001	
KELIM					
≥1	20,663	45/274	2.18	1	1
<1	9740	44/144	4.52	2.10 (1.38–3.18)	1.78 (1.16–2.72)
*p*-value				<0.001	0.008
Treatment response					
Responder	29,686	67/392	2.26	1	
Non-responder	716	22/26	30.73	19.00 (11.4–31.8)	
*p*-value				<0.001	
Histopathological type					
Others	22,841	57/305	2.50	1	1
HGSC	7562	32/113	4.23	1.71 (1.11–2.64)	1.13 (0.71–1.79)
*p*-value				0.015	0.608
NLR					
≤1.61	6657	9/87	1.35	1	1
>1.61	23,746	80/331	3.37	2.49 (1.25–4.97)	1.38 (0.66–2.87)
*p*-value				<0.001	0.393
PLR					
≤114.43	6865	8/86	1.17	1	1
>114.43	23,537	81/332	3.44	2.97 (1.44–6.15)	1.95 (0.91–4.18)
*p*-value				0.003	0.087
Time from surgery to start of chemotherapy					
≤21 days	18,193	56/253	3.08	1	
>21 days	12,210	33/165	2.70	0.87 (0.57–1.34)	
*p*-value				0.540	

HR: Hazard ratio; PDS: Primary debulking surgery; IDS: Interval debulking surgery; HGSC: High-grade serous carcinoma; KELIM: CA-125 ELIMination rate constant K; NLR: Neutrophil-to-lymphocyte ratio; PLR: Platelet-to-lymphocyte ratio.

## Data Availability

The original contributions presented in this study are included in the article. Further inquiries can be directed at the corresponding author.

## Abbreviations

CI	Confidence interval
EOC	Epithelial ovarian cancer
FIGO	Fédération Internationale de Gynécologie et d’Obstétrique
HR	Hazard Ratio
HGSC	High-grade serous carcinoma
HRD	Homologous Recombination Deficiency
IDS	Interval debulking surgery
KELIM	CA-125 elimination rate constant K
PDS	Primary debulking surgery
NLR	Neutrophil-to-lymphocyte ratio
PLR	Platelet-to-lymphocyte ratio
ROC	Receiver operating characteristic
